# Rapid Mandarin Tone Learning in Passive and Active Listening: A Magnetoencephalography Study

**DOI:** 10.1111/ejn.70487

**Published:** 2026-03-27

**Authors:** Kaijun Jiang, Qin Li, Jari L. O. Kurkela, Simo Monto, Jarmo A. Hämäläinen, Xueqiao Li, Piia Astikainen

**Affiliations:** ^1^ Jyväskylä Centre for Interdisciplinary Brain Research, Department of Psychology University of Jyväskylä Jyväskylä Finland

**Keywords:** foreign speech, magnetoencephalography, mismatch negativity, N2b, oddball condition

## Abstract

Adults can learn to represent foreign phonetic features, but only a few studies have tracked the phonetic learning process using both behavioral and brain activity measurements over several days. This study examined how 4 days of listening to changes in Mandarin tones on the vowel /a/ affect behavioral change detection and modulate magnetoencephalography (MEG) responses in native Finnish‐speaking adults (*n* = 9). On each day, participants completed one 25‐min passive listening session and one 45‐min active listening training session during which they responded behaviorally to tone changes. We found that behavioral accuracy improved immediately after the first day, and reaction times were faster on Days 3 and 4 compared with Day 1. In terms of brain activity, the amplitude of the M200 component, corresponding to the N2b in electroencephalography, increased during active listening from Day 1 to Days 2 and 3. No significant changes were observed in active listening in the M350 amplitude, which corresponds to the P3b component. During passive listening, an increase in the amplitude of the M215 response, corresponding to mismatch negativity, was observed after Day 1 and remained stable thereafter. These findings suggest that a single listening session of foreign speech sounds can modulate adults' behavioral and neural responses to phonetic changes, but replication with a larger sample is needed to confirm these effects.

## Graphical abstract

This study investigated how 4 days of passive and active listening to Mandarin tone changes in /a/ affected behavioral change detection and MEG responses in native Finnish‐speaking adults. Perceptual learning of foreign speech sounds was reflected in enhanced magnetic mismatch negativity and N2b as well as improved behavioral accuracy. All measures improved after one brief learning session.

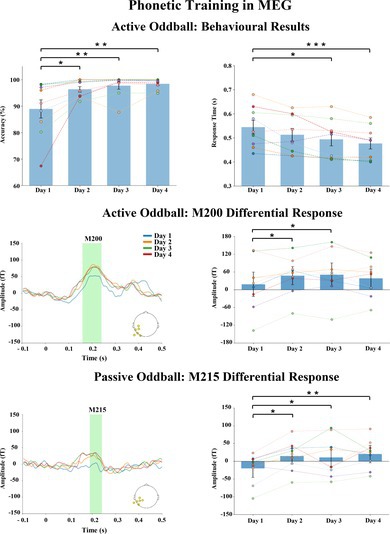

**Abbreviations**
ANOVA: analysis of variance
ECG: electrocardiogram
EEG: electroencephalography
EOG: electro‐oculogram
ERFs: event‐related fields
ERPs: event‐related potentials
ICA: independent component analysis
ISI: inter‐stimulus interval
MEG: magnetoencephalography
MMN: mismatch negativity
MMNm: magnetic mismatch negativity
tSSS: spatiotemporal signal space separation

## 1 Introduction

One of the fundamental cognitive tasks in early infancy is to accurately represent the speech sounds of the surrounding linguistic environment. Research has shown that young infants are sensitive to subtle acoustic distinctions across languages (Werker and Lalonde 1988). However, this broad sensitivity declines within the first year of life, as infants begin to specialize in the speech sounds of their native language (Cheour et al. 1998; Polka and Werker 1994). For adult foreign language learners, it is often challenging to distinguish non‐native speech sounds (Kuhl et al. 2008; Sparks and Ganschow 1993). Previous studies have shown that, although foreign speech sounds are often difficult to perceive accurately, perceptual training can enhance the perception of non‐native language features, and it remains effective across the lifespan (Callan et al. 2003). The present study investigates the time course of learning foreign vowel phonetic features over a short, four‐day training period using a change detection paradigm. Magnetoencephalography (MEG) was used to track day‐by‐day changes in neural responses associated with learning.

Change detection can be examined using the oddball paradigm, in which infrequent deviant stimuli are embedded within a sequence of standard stimuli. Electroencephalography (EEG) and MEG provide time‐resolved information about perceptual processing by capturing rapid neural responses to sensory stimuli (Kujala and Näätänen 2010; Näätänen et al. 2007; Sams et al. 1985). Mismatch negativity (MMN), or its magnetic counterpart (magnetic MMN, MMNm), is typically elicited in oddball conditions and reflects the brain's ability to detect changes in a sequence of auditory inputs (Näätänen 2001; Näätänen et al. 1978). MMN is typically elicited within 100–250 ms latency in response to deviations in auditory features such as frequency, duration, intensity, or phonemic content, even when attention is directed elsewhere (Näätänen et al. 2007; Sams et al. 1985). It is sometimes followed by P3a reflecting a preattentive shift of attention toward deviant sounds (Escera and Corral 2007). When participants actively attend to the stimuli, the N2b component of event‐related potentials (ERPs) typically emerges, and the MMN may be reduced in amplitude, overlap with N2b, or precede it (Alain et al. 2010; Folstein and Van Petten 2008; Näätänen et al. 1982; Sams et al. 1983). N2b is subsequently followed by P3b, which is associated with the conscious detection of stimulus changes and the updating of stimulus representations within attentional and perceptual context, consistent with predictive coding models of perception (for reviews, see Kok 2001; Polich 2007).

Previous studies have investigated phonetic learning with various auditory training methods, such as auditory discrimination training (e.g., Kraus et al. 1995; Tremblay et al. 1997), listen‐and‐repeat training (e.g., Tamminen et al. 2015), and other forms of listening‐based training (e.g., Ylinen et al. 2010; Zhang et al. 2009), using MMN and P3a components as neural indices of phonetic representations. These studies typically employed pre‐ and post‐training EEG recordings and demonstrated training‐induced increase in MMN amplitude in response to subtle speech sound contrasts, indicating phonetic learning. However, a key limitation is that these studies do not capture neural activity during the training itself, leaving the temporal dynamics of the learning process unexamined. The use of different types of training and test conditions also complicates the interpretation of results, as even preattentive test sessions can induce perceptual learning themselves (see Kurkela et al. 2019 for evidence of passive learning of foreign speech sound features).

To address these gaps, we investigated how repeated exposure to an oddball sequence of non‐native speech sounds, that is, vowel /a/ with Mandarin Chinese lexical tone variations, affects the dynamics of change detection, both behaviorally and neurally. Rather than using a traditional pre–post measurement design, MEG recordings were conducted over four consecutive days. Each day began with a passive listening session, followed by an active listening session in which participants responded via button press whenever they detected a tone change. The inclusion of both passive and active listening allowed us to examine distinct yet complementary aspects of auditory learning. Passive listening indexed preattentive neural plasticity—automatic sensitivity to sound changes that occur without focused attention—whereas active listening engaged attentional and decision‐related mechanisms that contribute to conscious detection and task performance. By tracking both processes across days, we aimed to determine whether learning‐related changes in neural responses emerge first at a preattentive level or require active attention, and whether these processes develop in parallel or follow different time courses. This longitudinal design thus enabled a detailed characterization of the neural dynamics underlying rapid auditory–phonetic learning.

In our previous study, we used a pre–post measurement design and ERP recordings to investigate the effects of passive listening to repetitions of the vowel /a/ featuring changes in Mandarin lexical tones (Kurkela et al. 2019). Eight hours of passive exposure, divided into two‐hour daily sessions over four consecutive days, increased the amplitudes of the P3a and P3b components, indicating enhanced efficiency of preattentive attentional shifts toward tonal changes. However, no significant increase in MMN and N2b amplitude was observed. Notably, neural changes likely preceded the behavioral changes, as change detection accuracy did not improve following passive exposure.

Here, we build on our previous study (Kurkela et al. 2019) and further investigate, using MEG, how active listening to Mandarin tones enhance brain activity and behavioral detection. We expect that the 4‐day training will result in behavioral learning and modulation of event‐related fields (ERFs), reflecting improvements in both pre‐attentive and attentive change detection (corresponding to MMN and N2b, respectively, in ERPs) and attention orienting toward changes (P3 in ERPs; Polich 2007). The exposure time is shorter than in our previous study, which involved passive exposure with 120‐min daily sessions (here, 25 min of passive and 45 min of active listening). Still, because active training is included, we expect that changes in both behavioral performance (increased accuracy and shortened reaction time) and brain activity (a change in differential response, i.e., deviant—standard, amplitude) across training days will occur.

We recently analyzed, using a single‐trial machine learning method, data recorded during passive exposure in Kurkela et al. (2019). We observed neural changes already after the first two hours of training (Li et al. 2025), suggesting that auditory representations can adapt rapidly to novel phonetic contrasts. However, longitudinal studies that track phonetic learning on a daily basis remain rare, and because the study by Kurkela et al. (2019) did not include active training with behavioral responses, it is unclear whether neural and behavioral changes unfold in parallel or follow different time courses. We hypothesized that active training would elicit early neural changes similar to those observed after passive exposure, but that repeated daily sessions may further strengthen these effects and be accompanied by corresponding improvements in behavioral performance.

## 2 Method

### 2.1 Participants

Eleven monolingual Finnish‐speaking participants volunteered for the study during its data collection period. A priori power analysis was conducted in G*Power (Version 3.1.9.7) for a repeated‐measures ANOVA with one within‐subjects factor (four levels). A large effect size (*f* = 0.40, Cohen 2013) was assumed, *α* = 0.05, correlation among repeated measures = 0.50, and nonsphericity correction *ε* = 1. The analysis indicated that a minimum of 10 participants would be required to achieve 80% power (1—*β* = 0.80) to detect a large within‐subject effect at *p* < 0.05. This number of participants is comparable to that in previous EEG studies investigating foreign speech sound learning (Hisagi et al. 2016; Kaan et al. 2007; Menning et al. 2002; Tamminen et al. 2024; Tremblay et al. 1998; Tremblay and Kraus 2002).

The participants were recruited through announcements posted on notice boards and distributed via email lists at the University of Jyväskylä. Participants were informed during recruitment that the study involved 4 days of training on Mandarin Chinese tones. The inclusion criteria for the study were: age of 18–30 years, right‐handed, normal hearing as measured by audiometry, and self‐reported normal vision (or corrected‐to‐normal vision). The exclusion criteria for the study were neurological or psychiatric disorders, including sleep problems, and exposure to or training in tonal languages (except exposure during a maximum of a 2‐week trip to countries where tonal languages are spoken). Written informed consent was obtained from each participant before the study. The experiment was conducted according to the Declaration of Helsinki, and the ethical committee of the University of Jyväskylä approved the research protocol.

Nine participants' data (five females, three males, and no gender information for one participant; mean age = 21.13 years, standard deviation = 2.85) were included in the analysis because two participants' data were missing from one or more training sessions due to a technical issue. Five participants reported having some musical training or music as a hobby, but none were professional musicians.

### 2.2 Stimuli

Vowel /a/ with different tones was presented to participants binaurally during the listening sessions through plastic tubes and earpieces using an MEG‐compatible high‐fidelity sound system. Because participants were native Finnish speakers and tone is not a lexical feature in the Finnish language, this feature was foreign to them. The sounds were prepared so that first, the phoneme/a/was spoken by a female native Mandarin Chinese speaker with rising (i.e., Chinese lexical tone 2) and falling (i.e., Chinese lexical tone 4) pitch contour, and they were recorded at a sampling rate of 44.1 kHz. The sounds were then digitally edited using SoundForge software (SoundForge 9, Sony Corporation, Japan) to modify their duration to 200 ms. A pitch tier transfer was performed using Praat software to isolate the lexical tones and keep the rest of the acoustic features identical (Praat v5.4.06, University of Amsterdam). The pitch tier transfer generated a rising tone and a falling tone, which were similar except for a pitch contour difference in fundamental frequency (F0). These two tones were taken as the endpoint stimuli to create a continuum of lexical tones with 10 interval steps. A morphing technique was performed in MATLAB (MathWorks Inc., MA, US), and a STRAIGHT tool (Kawahara et al. 1999) was used to create the tones applied in the experiment. All stimuli were normalized to have the same root mean square intensity. The detailed procedure for the stimulus preparation has been reported elsewhere (Xi et al. 2010).

**FIGURE 1:**
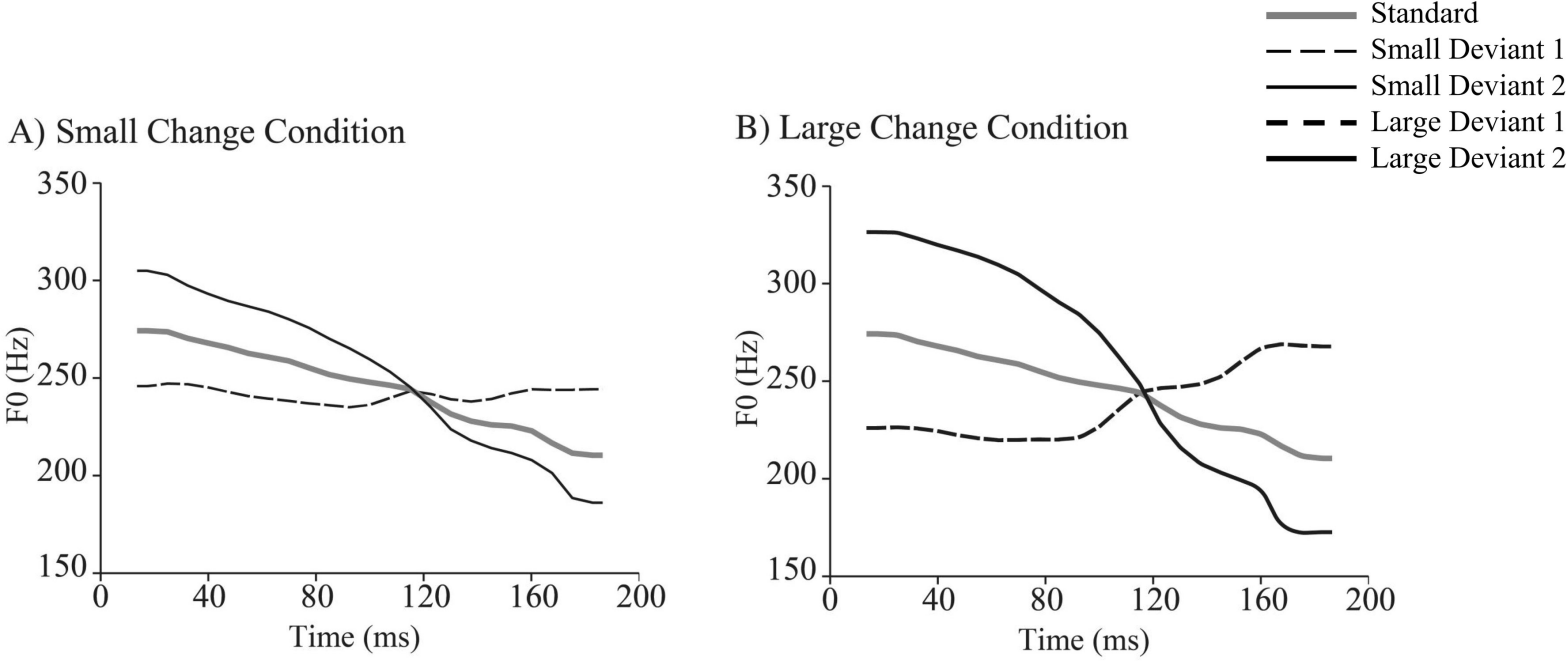
Stimuli in the Small and Large change conditions. The responses to Deviant 1 and Deviant 2 within each condition were averaged for the analysis. Please note that the duration of all stimuli is 200 ms, although the F0 track does not extend to the waveform onset and offset because pitch was estimated using frame‐based, center‐aligned analysis windows.

In the present study, two levels of changes (Large and Small change conditions, Figure 1) were presented in separate oddball conditions. The Small and Large change conditions were included to prevent potential ceiling or floor effects that might occur if only highly distinct or overly subtle tone contrasts were used. The repeatedly presented standard sound was a falling tone (Token 7 in Xi et al. 2010). Deviant sounds in the Small change condition were tones with a small physical difference from the standard tone (corresponding to Tokens 5 and 9 in Xi et al. 2010), whereas deviant sounds in the Large change condition were tones with a physically larger difference from the standard tone (corresponding to Tokens 3 and 11 in Xi et al. 2010). The frequently presented (80%) standard stimulus was randomly interspersed with two rare deviant stimuli (Deviant 1 and Deviant 2, 10% for each; Figure 1), with a sound pressure level (SPL) of 70 dB. Inter‐stimulus interval (ISI) randomly varied from 450 to 550 ms (offset to onset). Stimulus presentation was controlled by the Presentation software (Neurobehavioral Systems Inc., Albany, CA, United States).

### 2.3 Procedure

The MEG recordings were conducted on four consecutive days. Each participant was assigned a consistent daily time slot (morning, noon, or afternoon), and all recordings were conducted at approximately the same time each day (within a two‐hour window). Each day began with a passive listening session, in which participants first listened to the Large change block followed by the Small change block while watching a silent movie. This was followed by the active session, in which participants completed two Large change blocks followed by two Small change blocks and were instructed to press a button whenever they detected a deviant stimulus. This order ensured that training always started with easier, more detectable stimuli. Each stimulus block contained 1000 stimuli, with short breaks between blocks. The passive and active listening sessions lasted approximately 25 and 45 min per day, respectively (excluding breaks). The passive session was deliberately shorter to maintain participant engagement and to focus primarily on the effects of active training, while still providing a complementary measure of preattentive auditory processing.

### 2.4 MEG Measurement

The MEG recordings occurred in a magnetically shielded room at the University of Jyväskylä's MEG Laboratory. The evoked magnetic fields were captured using a whole‐head TRIUX system (Megin Oy, Helsinki, Finland), with 306 sensors and a bandpass of 0.1–330 Hz and a sampling rate of 1000 Hz. In addition, blinks and eye movements were recorded with an electro‐oculogram (EOG), with electrodes placed above and below the right eye (the vertical EOG) and on the outer canthi of both eyes (the horizontal EOG). Heartbeats were recorded with an electrocardiogram (ECG) with two electrodes placed on the chest. Before the experiment, the positions of three anatomical landmarks (the nasion and the left and right preauricular points), five head‐position indicator coils (two at the temples, two behind the ears, and one on the forehead), and additional scalp points were recorded using a 3D digitizer (Fastrak, Polhemus, Vermont, USA). During the recordings, participants were seated in a 68° upright gantry position with their heads inside the helmet‐shaped magnetometer.

### 2.5 Analysis of Behavioral and Neuronal Data

The behavioral responses from the active listening condition were analyzed. Responses that occurred between 100 and 1000 ms after the offset of the deviant sound were included in the analysis for accuracy and response time.

For the MEG, data from 102 magnetometers were selected for the analysis. The spatiotemporal signal space separation (tSSS) method (Taulu and Hari 2009) in the MaxFilter software (Elekta‐Neuromag) was initially applied to eliminate external interference. MaxFilter software was also used to compensate for head movement and align the head positions across all participants and sessions. MNE‐Python Version 1.6.1 was used for further MEG analysis. Data were first loaded and subjected to notch filtering at 50, 100, 150, and 200 Hz to remove power‐line interference and its harmonics. Muscle artifacts were automatically annotated using the z‐score‐based method from MNE‐Python, with a Z‐score threshold of 10 and a filter frequency band between 110 and 140 Hz (Muthukumaraswamy 2013). EOG and ECG channels were explicitly set for artifact detection (Dammers et al. 2008). The continuous data were down‐sampled to 200 Hz and then band‐pass filtered between 1 and 40 Hz, effectively isolating the frequency bands of interest for further study and reducing unwanted low‐frequency drift and high‐frequency noise. The independent component analysis (ICA) was used for artifact removal in the data (Hyvärinen and Oja 2000), leveraging the FastICA algorithm (Ablin et al. 2018; Hyvärinen 1999) due to its computational efficiency in separating independent sources and removing eye movement‐related and cardiac artifacts. After applying ICA, the data were segmented into epochs starting at 100 ms before and ending 600 ms after stimulus onset. Baseline correction was applied by subtracting the average response amplitude from −100 to 0 ms before stimulus onset from all data points within each epoch (Gramfort et al. 2014).

For each participant, trials were averaged separately for the active and passive listening conditions, for the Large change and Small change conditions, and for each stimulus type (standard and deviant; Deviant 1 and Deviant 2 responses within the Small and Large change conditions were averaged, see Figure 1). Then, the difference wave between the deviant and standard responses (deviant minus standard) was calculated separately for each participant.

Based on prior EEG findings of Kurkela et al. (2019) and the grand‐averaged responses, time windows and sensors were selected for the analysis. The selected time windows for the active listening were 160–240 ms post‐stimulus for the M200, and 310–390 ms post‐stimulus for the M350, corresponding to N2b and P3b in ERPs, respectively (Kurkela et al. 2019). In the passive listening condition, time windows of 140–190 ms and 190–240 ms after stimulus onset were selected for M165 and M215, respectively, which possibly correspond to the MMN component. Two analysis windows were selected for MMN based on visual inspection of the grand‐averaged differential responses, which revealed two distinct peaks. These windows were analyzed separately to capture temporally distinct response components. No clear P3a‐like activity was observed in the data. The sensors for M200 were MEG1621, MEG1631, MEG1811, MEG1641, MEG0441, MEG1911; for M350 were MEG1841, MEG1631, MEG1911, MEG2011, MEG1831, MEG1821; and for M165 and M215 were MEG1621, MEG1811, MEG1821, MEG1611, MEG1631, MEG0441. The sensors are indicated in the figures reporting the MEG results.

### 2.6 Statistics

All statistical analyses were conducted using JASP (Version 0.18.3). A repeated‐measures analysis of variance (ANOVA) with the within‐subjects factors Day (1–4) and Stimulus type (Small change, Large change) was used to examine changes in behavioral accuracy, response time, and brain responses. Whenever Mauchly's test indicated that the assumption of sphericity was violated, degrees of freedom were corrected using Greenhouse–Geisser estimates of sphericity (when *ε* < 0.75) or Huynh–Feldt estimates of sphericity (when *ε* > 0.75). Post hoc pairwise comparisons (*t*‐tests, 2‐tailed) were Bonferroni‐corrected.

In addition to frequentist analyses, Bayesian repeated‐measures ANOVAs and Bayesian paired‐samples *t*‐tests were conducted in JASP using default priors. Bayes factors (*BF*_10_) were reported to quantify evidence for or against the presence of an effect. Frequentist and Bayesian outcomes were interpreted jointly. Significant *p*‐values indicated reliable effects in the frequentist framework, whereas Bayes factors were used to classify evidence as supporting the null hypothesis (*BF*_10_ < 1/3), supporting the alternative hypothesis (*BF*_10_ > 3), or providing inconclusive evidence (*BF*_10_ between 1/3 and 3), following Jeffreys' conventions (Jeffreys 1961). In cases where the *p*‐value and *BF*_10_ diverged (e.g., a nonsignificant *p*‐value with *BF*_10_ > 3, or vice versa), these discrepancies were explicitly noted and interpreted with caution.

For all tests, a *p*‐value smaller than 0.05 was considered significant. Partial eta squared (*η*_*p*_^*2*^) and Cohen's *d* were used as effect size estimates. *η*_*p*_^*2*^ values of 0.01, 0.06, and 0.14 indicated small, medium, and large effects, respectively, whereas Cohen's *d* values of 0.20, 0.50, and 0.80 represented small, medium, and large effects (Cohen 1992). Degrees of freedom (df) are reported as uncorrected.

## 3 Results

### 3.1 Behavioral Results in Active Listening

**FIGURE 2:**
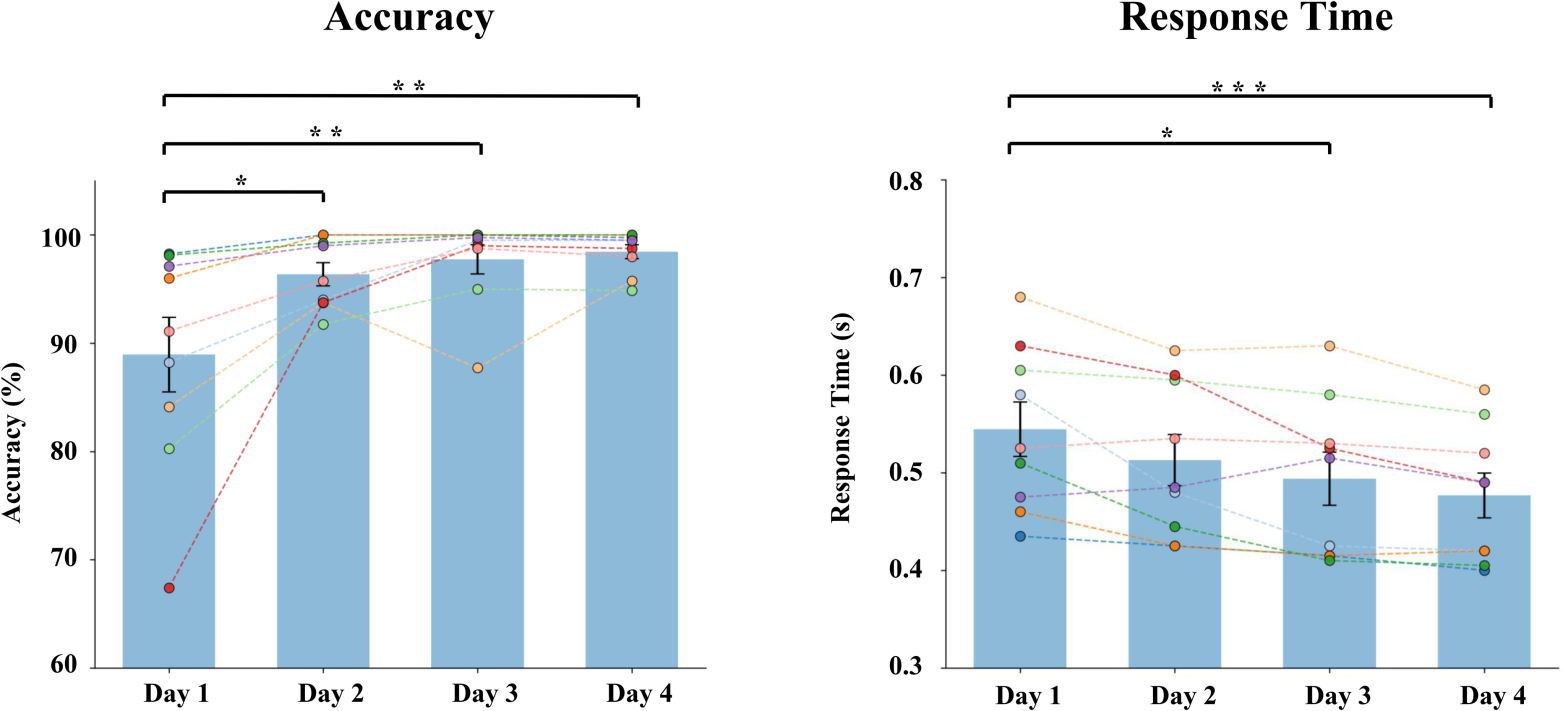
Behavioral results. Accuracy and response time of behavioral change detection in active listening averaged over the Small and Large change conditions and the two deviant stimulus responses. The error bars represent the standard error of mean. Each dot represents the value of an individual subject, while dashed lines connect repeated measurements from the same subject across days. *p < 0.05, **p < 0.01, ***p < 0.001.

The results of the two‐way repeated measures ANOVA with the within‐subjects variables Day and Stimulus type are reported in Table 1. The main effect of Day was significant for both accuracy and response time. The results of the post hoc pairwise comparisons for the main effect of Day are shown in Table 2 and in Figure 2.

**TABLE 1:** Behavioral results: Two‐way repeated measures ANOVA.

Variable	Effect	df	*F*	*p*	*η*_*p*_^*2*^	*BF*_10_
Accuracy	Day	3, 24	7.62	**0.018**	0.49	23.96
	Stimulus type	1, 8	0.01	0.923	< 0.01	0.27
	Day × stimulus type	3, 24	0.11	0.955	0.01	0.16
Response time	Day	3, 24	8.24	**0.013**	0.51	35.90
	Stimulus type	1, 8	0.30	0.597	0.04	0.32
	Day × stimulus type	3, 24	0.10	0.883	0.01	0.18

*Note:* Significant *p*‐values (*p* < 0.05) are marked in bold.

**TABLE 2:** Behavioral results: Post hoc pairwise comparisons for the main effect of Day.

Variable	Comparison	df	*t*	*p*	Cohen's *d*	*BF*_10_
Accuracy	Day 1 vs. Day 2	8	3.31	**0.018**	1.20	21.60
	Day 1 vs. Day 3	8	3.93	**0.004**	1.42	22.54
	Day 1 vs. Day 4	8	4.24	**0.002**	1.54	47.37
	Day 2 vs. Day 3	8	0.62	1.000	0.23	0.85
	Day 2 vs. Day 4	8	0.94	1.000	0.34	59.63
	Day 3 vs. Day 4	8	0.32	1.000	0.12	0.41
Response time	Day 1 vs. Day 2	8	2.22	0.219	0.40	20.09
	Day 1 vs. Day 3	8	3.54	**0.010**	0.63	14.75
	Day 1 vs. Day 4	8	4.74	**< 0.001**	0.85	158.56
	Day 2 vs. Day 3	8	1.32	1.000	0.24	1.56
	Day 2 vs. Day 4	8	2.53	0.111	0.45	28.93
	Day 3 vs. Day 4	8	1.21	1.000	0.22	17.55

*Note: p*‐values are Bonferroni‐corrected. Significant *p*‐values (*p* < 0.05) are marked in bold.

### 3.2 MEG Results in Active Listening

#### 3.2.1 M200 (N2b)

**FIGURE 3:**
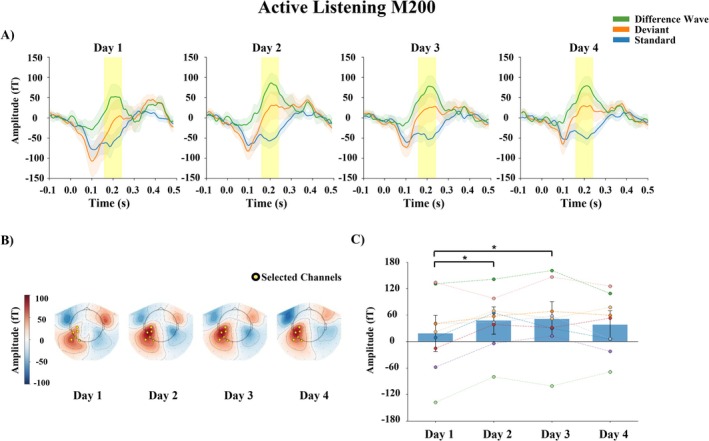
M200 (N2b) in active listening. Grand‐averaged responses averaged over the Small and Large change conditions. (A) Waveforms for standard and deviant stimuli and difference waves (deviant − standard) from selected channels (averaged over the sensors marked in the topographies); shaded areas represent 95% confidence interval. (B) Topographic maps of the mean differential activity at a time window of 160–240 ms after stimulus onset. (C) The bars represent the mean amplitude of the differential activity each day, averaged over the sensors. The error bars represent the standard error of mean. Each dot represents the value of an individual subject, while dashed lines connect repeated measurements from the same subject across days. *p < 0.05.

The results of the two‐way repeated measures ANOVA are reported in Table 3. A main effect of Day was found. The results of the post hoc pairwise comparisons for the main effect of Day are shown in Table 4. The amplitude of M200 was larger on Day 2 and Day 3 than on Day 1. The grand‐averaged responses of M200 averaged over the Small and Large change conditions are shown in Figure 3.

**TABLE 3:** M200: Results from the two‐way repeated measures ANOVA.

Effect	df	*F*	*p*	*η*_*p*_^*2*^	*BF*_10_
Day	3, 24	4.23	**0.015**	0.35	1.36
Stimulus type	1, 8	< 0.01	0.961	< 0.01	0.26
Day × stimulus type	3, 24	0.24	0.869	0.03	0.13

*Note:* Significant *p*‐values (*p* < 0.05) are marked in bold.

**TABLE 4:** M200: Post hoc pairwise comparisons for the main effect of Day.

Comparison	df	*t*	*p*	Cohen's *d*	*BF*_10_
Day 1 vs. Day 2	8	2.90	**0.047**	0.38	8.85
Day 1 vs. Day 3	8	3.24	**0.021**	0.43	7.75
Day 1 vs. Day 4	8	1.99	0.351	0.26	0.81
Day 2 vs. Day 3	8	0.34	1.000	0.05	0.26
Day 2 vs. Day 4	8	0.92	1.000	0.12	0.36
Day 3 vs. Day 4	8	1.26	1.000	0.17	0.50

*Note: p*‐values are Bonferroni‐corrected. Significant *p*‐values (*p* < 0.05) are marked in bold.

#### 3.2.2 M350 (P3b)

**FIGURE 4:**
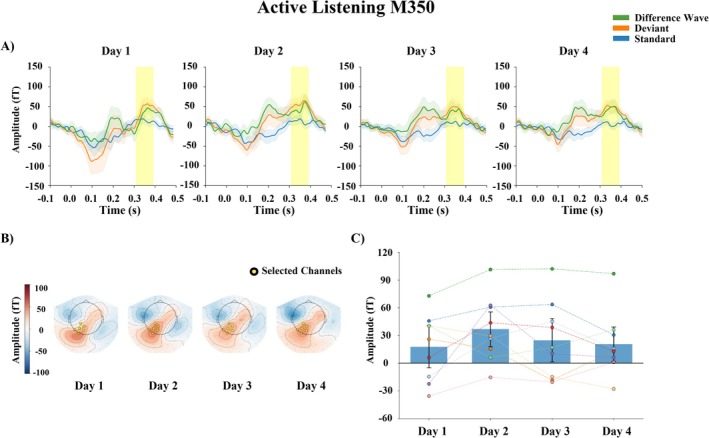
M350 (P3b) in active listening. Grand‐averaged responses averaged over the Small and Large change conditions. (A) Waveforms for standard and deviant stimuli and difference waves (deviant − standard) from selected channels (averaged over the sensors marked in the topographies); shaded areas represent 95% confidence interval. (B) Topographic maps of the mean differential activity at a time window of 310–390 ms after stimulus onset. (C) The bars represent the mean amplitude of the differential activity each day, averaged over the sensors. The error bars represent the standard error of mean. Each dot represents the value of an individual subject, while dashed lines connect repeated measurements from the same subject across days. No significant amplitude changes were observed.

The results of the two‐way repeated measures ANOVA are reported in Table 5. No significant effects were found. The grand‐averaged responses of M350 averaged over the Small and Large change conditions are shown in Figure 4.

**TABLE 5:** M350: Results from the two‐way repeated measures ANOVA.

Effect	df	*F*	*p*	*η*_*p*_^*2*^	*BF*_10_
Day	3, 24	1.29	0.302	0.14	0.22
Stimulus type	1, 8	1.06	0.333	0.12	0.39
Day × stimulus type	3, 24	0.98	0.383	0.11	0.14

### 3.3 MEG Results in Passive Listening

**FIGURE 5:**
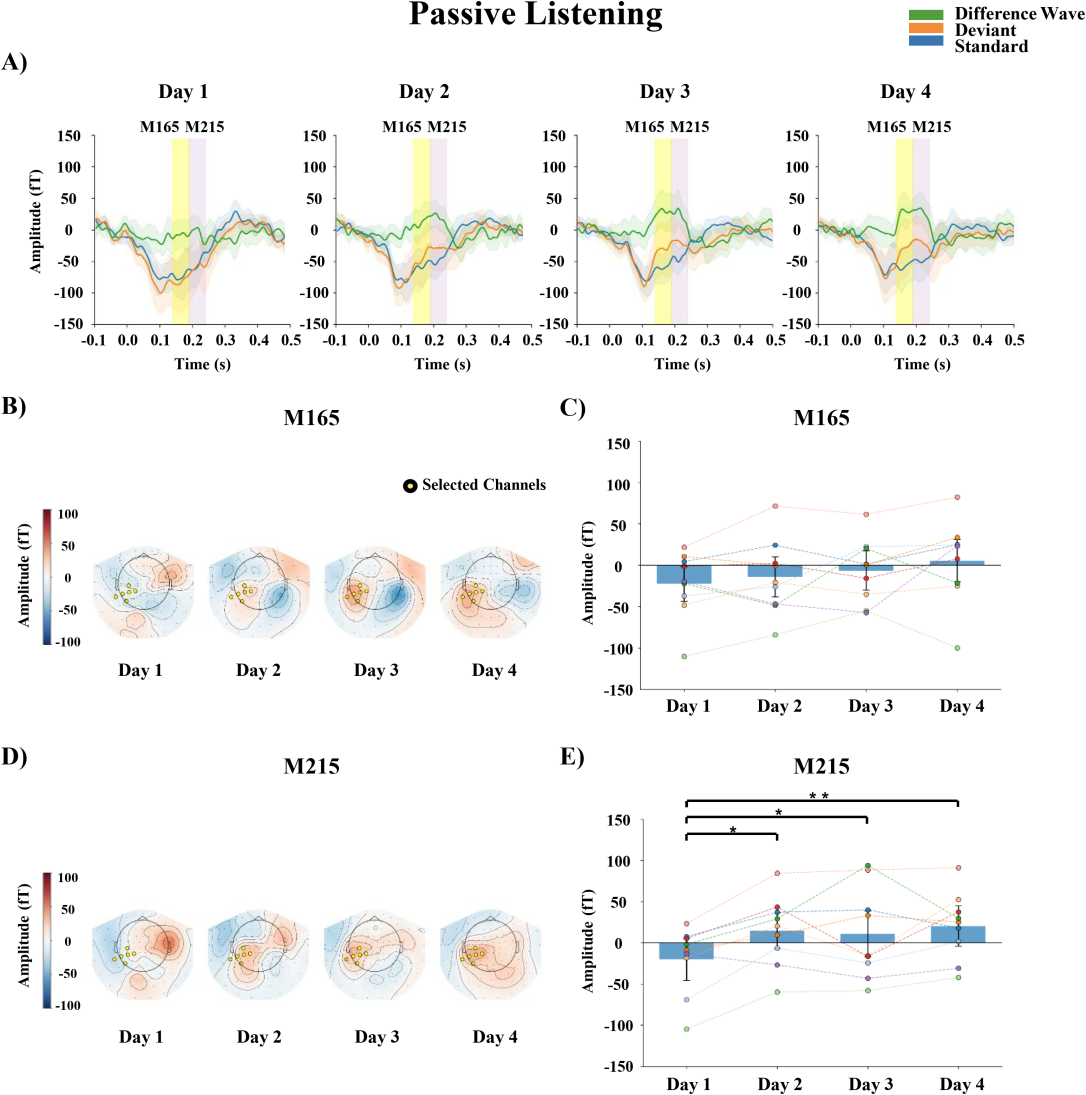
Results in passive listening. Grand‐averaged responses averaged over Small and Large change conditions. (A) M165 and M215 (MMN) waveforms for standard and deviant stimuli and difference waves (deviant − standard) from selected channels (averaged over the sensors marked in the topographies); shaded areas represent 95% confidence interval. (B) Topographic maps showing the mean differential activity for M165 at 140–190 ms after stimulus onset. (C) Mean amplitude of the differential activity for M165 each day, averaged over the sensors. Note that no significant amplitude changes were observed for M165. (D) Topographic maps showing the mean differential activity for M215 at 190–240 ms after stimulus onset. (E) Mean amplitude of the differential activity for M215 on each day, averaged over the sensors. The error bars in (C) and (E) represent the standard error of the mean, and each dot represents the value of an individual subject, while dashed lines connect repeated measurements from the same subject across days. *p < 0.05, **p < 0.01.

The grand‐averaged responses averaged for M165 and M215 (MMN) over the Small and Large change conditions are shown in Figure 5.

#### 3.3.1 M165

The results of the two‐way repeated measures ANOVA are reported in Table 6. No significant main effects or interaction effects were found.

**TABLE 6:** M165: Results from the two‐way repeated measures ANOVA.

Effect	df	*F*	*p*	*η*_*p*_^*2*^	*BF*_10_
Day	3, 24	2.69	0.069	0.25	0.60
Stimulus type	1, 8	5.03	0.055	0.39	1.10
Day × stimulus type	3, 24	0.30	0.827	0.04	0.23

#### 3.3.2 M215

The results of the two‐way repeated measures ANOVA are reported in Table 7. A main effect of Day was found. The post hoc pairwise comparisons for the main effect of Day showed that the amplitude increased from Day 1 to Days 2–4 (Table 8).

**TABLE 7:** M215: Results of two‐way repeated measures ANOVA.

Effect	df	*F*	*p*	*η*_*p*_^*2*^	*BF*_10_
Day	3, 24	5.69	**0.004**	0.42	3.57
Stimulus type	1, 8	0.24	0.639	0.03	0.35
Day × stimulus type	3, 24	0.57	0.642	0.07	0.27

*Note:* Significant *p*‐values (*p* < 0.05) are marked in bold.

**TABLE 8:** M215: Post hoc pairwise comparisons for the main effect of Day.

Comparison	df	*t*	*p*	Cohen's *d*	*BF*_10_
Day 1 vs. Day 2	8	3.23	**0.021**	0.65	29.87
Day 1 vs. Day 3	8	2.89	**0.048**	0.58	1.86
Day 1 vs. Day 4	8	3.77	**0.006**	0.76	39.46
Day 2 vs. Day 3	8	0.34	1.000	0.07	0.26
Day 2 vs. Day 4	8	0.54	1.000	0.11	0.29
Day 3 vs. Day 4	8	0.88	1.000	0.18	0.32

*Note:* *p*‐values are Bonferroni‐corrected. Significant *p*‐values (*p* < 0.05) are marked in bold.

## 4 Discussion

This study investigated phonetic learning of foreign Mandarin tones in adults over a four‐day exposure period using a change detection paradigm. Behavioral and neural changes were tracked under both active and passive listening conditions, allowing us to map the time course of learning. We observed improvements in behavioral change detection performance and corresponding brain activity as early as the first day, highlighting rapid neural plasticity during the early stages of phonetic acquisition.

Behavioral results showed higher accuracy in detecting changes on Days 2–4 compared with Day 1, with Bayesian analysis consistently supporting these differences (*BF*_10_ > 10). Although some comparisons were not significant according to *p*‐values (e.g., Day 2 vs. Day 4), Bayesian analysis still provided strong evidence for differences (*BF*_10_ > 20). Overall, behavioral accuracy improved after the first day, and it is possible to interpret that accuracy increased from the second day onward. The change detection task appeared relatively easy, as the mean accuracy on Day 1 was already close to 90%, leaving limited room for improvement. Response times showed a gradual improvement over days. Although significant reductions compared with Day 1 were observed only on Days 3 and 4, Bayesian analysis suggested meaningful improvements starting on Day 2 (*BF*_10_ > 20). These findings align with previous categorization training research demonstrating that adults can enhance their discrimination of non‐native speech contrasts through short‐term training, typically involving multiple sessions conducted over one to three weeks (e.g., Lively et al. 1993; Perrachione et al. 2011). Although these studies provide compelling evidence for phonetic learning, they often rely on pre‐ and post‐training assessments, leaving the time course of learning underexplored. In contrast, the present study employed a change detection paradigm with daily behavioral and neural measurements across four consecutive days. This design allowed us to capture the dynamics of phonetic learning in greater detail. This approach is also advantageous, as using different training and testing conditions—such as categorization during training and discrimination during testing—can complicate the interpretation of learning effects due to task‐specific influences.

In addition to behavioral changes, neural changes emerged rapidly. During active change detection, the M200 component increased significantly from Day 1 to the subsequent days. Although the main effect of Day showed a large effect size (partial eta‐squared), the corresponding Bayes factors (*BF*_10_ between 1 and 3) provided only anecdotal support for the alternative hypothesis. In contrast, the post hoc pairwise comparisons revealed clear differences between Day 1 and Day 2 as well as between Day 1 and Day 3, and the corresponding *BF*_10_ values exceeded 3 (*BF*_10_ > 7), providing substantial evidence for these learning‐related changes. Given its latency and topographical distribution, the M200 likely reflects early phonetic processing and auditory discrimination, corresponding to the N2b component observed in ERP studies (Näätänen and Gaillard 1983).

Surprisingly, no significant changes were observed in the M350 amplitude, which corresponds to the P3b component in ERPs research and is typically associated with attentional allocation (Polich 2007). The pattern of results during active listening differs from our previous study (Kurkela et al. 2019), where the exposure was based on passively listening to Mandarin tones in an oddball paradigm, and the learning related changes were investigated with pre‐ and post‐measurements. That is, the passive exposure led to an increase in P3b—but not in N2b—amplitude in the active test condition, and no behavioral improvement was found in change detection. This dissociation may suggest that active engagement in phonetic training may preferentially enhance earlier perceptual processing stages (indexed by M200/N2b), whereas passive exposure might primarily influence later, attention‐related processes (indexed by M350/P3b); however, more investigation is needed to confirm this. It is also possible that the relatively easy change detection task, together with the attentive training, produced a ceiling effect, such that the cognitive processes associated with the P3b were already maximally engaged, leading to no observable changes in its amplitude.

No differences were observed between the two active listening blocks within each day, either in the behavioral results or in the neural responses. Such a lack of within‐day block effects may hint that learning‐related changes occurred mostly during nocturnal sleep, consistent with evidence that sleep plays a vital role in the consolidation and stabilization of speech learning (Fenn et al. 2003).

For passive listening, our findings align with previous studies demonstrating that the amplitude of MMN increases following phonetic training, typically conducted over several days or weeks, reflecting plasticity in the central auditory system (e.g., Kraus et al. 1995; Menning et al. 2002). Here, two analysis windows were applied because two separate peaks were observed in the MMN time course (M165 and M215). Learning‐related effects were evident in the later component (M215) but not the earlier one (M165), suggesting that short‐term training preferentially modulates later stages of auditory processing, whereas earlier responses perhaps reflect more automatic sensory processes. It should be emphasized, however, that the functional significance of these two components is not yet clear, and we cannot determine which specific auditory processes are reflected in the early versus later peaks.

The MMN (M215, but not M165) increased in amplitude from Day 1 to subsequent days, with a clear rise by Day 2 and a strong difference maintained through Day 4. No meaningful differences were observed among the later days themselves. Although the Day 1 vs. Day 3 comparison showed only anecdotal Bayesian evidence, the increase from Day 1 to Day 4 was supported by both a small *p*‐value and a strong Bayes factor, indicating that the primary change occurred after the first day and was sustained. The fast neural changes as indexed by the MMN amplitude were not surprising. Enhanced MMN amplitudes have also been reported after just a few minutes to several hours of training, suggesting that rapid neural plasticity can emerge within a short timescale (for non‐speech sound patterns, see Atienza et al. 2002; Gottselig et al. 2004, for Mandarin tones, see Li et al. 2025).

In our previous study (Kurkela et al. 2019), which also employed a four‐day training protocol (2 h per day) in a fully passive setting, no changes were observed in MMN amplitude or in behavioral responses, although an increase in P3a amplitude was detected. In contrast, the present MEG study did not reveal a clear P3a‐like component in the grand‐averaged waveforms. It remains unclear whether EEG and MEG differ in their sensitivity to evoked responses to speech sound changes, or whether other methodological differences between the studies account for the discrepancy. Taken together, findings from the present and our previous study (Kurkela et al. 2019) suggest that active training is substantially more effective than passive exposure in promoting phonetic change detection both at behavioral and neural levels. Some previous studies have suggested that neural changes may precede observable behavioral improvements during auditory learning (Tremblay et al. 2009). In the present study, however, changes in neural responses, specifically in the M215 (MMN) during passive listening and in the M200 (N2b) during active listening, were observed concurrently with improvements in behavioral accuracy already after the first day of training. This temporal alignment may reflect the relatively low perceptual demands of the change detection task.

When interpreting the results, it should be emphasized that the present study cannot provide conclusive evidence for either phonetic (acoustic) or phonemic learning. Behavioral performance and neural responses improved rapidly over the course of training, which is consistent with rapid phonetic plasticity rather than the slower formation of phonemic categories. At the same time, participants were explicitly informed that the task involved perceiving Mandarin tones, and the stimuli included contrasts related to tonal categories. Although the sounds consisted of isolated vowels rather than full syllables or words, this instruction may have encouraged listeners to partially map the sounds onto speech categories, allowing for limited phonemic learning. Overall, although partial mapping onto phonemic categories cannot be excluded, the observed learning pattern appears most consistent with rapid auditory plasticity at the phonetic level.

Although the present results provide evidence for Mandarin tone learning by Finnish‐speaking participants, they may not generalize to all language groups and language features. Both the foreign speech features and the participants' native language are likely to influence the learning outcomes (e.g., Kaan et al. 2007). Also, individual differences affect phonetic learning. For example, high‐variability training was effective only for participants with strong perceptual skills, whereas those with weaker perceptual abilities were negatively affected by the variability in the stimulus materials (Perrachione et al. 2011).

This study has some limitations, the most notable being the small sample size; therefore, the results should be interpreted with caution. However, the observed effects were relatively robust and consistently evident in both behavioral performance and MEG response. However, replication with a larger sample would strengthen the conclusions. Another limitation of our study is that the effects of passive and active listening could not be disentangled. It is possible that passive listening reflected cumulative learning from previous active sessions across the four‐day training period. Therefore, changes in M215 (MMN) are not directly comparable to those that could be observed during passive exposure alone. In addition, improvements in behavioral measures could partially reflect general task‐related or procedural learning effects, rather than purely perceptual changes. In contrast, the observed alterations in brain activity are less likely to be explained by such non‐specific factors, supporting their interpretation as neural markers of phonetic learning.

## 5 Conclusion

In summary, the findings demonstrate rapid and robust changes in both behavioral performance and neural plasticity associated with phonetic learning of foreign speech features. Neural changes were specifically linked to brain activity related to change detection—namely, components corresponding to the MMN and N2b identified in previous EEG studies—but not to later mechanisms typically associated with attentional shifting (P3b). Notably, these changes appeared immediately after the first day, demonstrating the effectiveness of attentive, change‐detection–based training in facilitating the learning of non‐native speech sound features.

## Author Contributions

**Kaijun Jiang:** formal analysis (lead), methodology (equal), visualization (lead), writing – original draft (lead), writing – review and editing (equal). **Qin Li:** data curation (equal), formal analysis (supporting), methodology (supporting). **Jari L. O. Kurkela:** conceptualization (equal), data curation (equal), investigation (lead), methodology (equal), writing – review and editing (supporting). **Simo Monto:** methodology (supporting), writing – review and editing (supporting). **Jarmo A. Hämäläinen:** conceptualization (equal), writing – review and editing (supporting). **Xueqiao Li:** data curation (equal), investigation (supporting), supervision (supporting), visualization (supporting), writing – review and editing (supporting). **Piia Astikainen:** conceptualization (equal), funding acquisition (lead), project administration (lead), resources (lead), supervision (lead), writing – review and editing (equal).

## Funding

The study was supported by the Research Council of Finland (Grant Numbers 351009 and 273134 for Piia Astikainen) and the Ellen and Artturi Nyyssönen Foundation (personal grant for Kaijun Jiang).

## Ethics Statement

The ethical committee of the University of Jyväskylä approved the research protocol. The experiment was undertaken in accordance with the Declaration of Helsinki.

## Consent

Written informed consent was obtained from all participants before their participation.

## Conflicts of Interest

The authors declare no conflicts of interest.

## Supporting information


**Table S1:** Behavioral results: Paired‐samples *t*‐tests comparing responses in the first and second stimulus blocks on each day for the Large change condition.
**Table S2:** Behavioral results: Paired‐samples *t*‐tests comparing responses in the first and second stimulus blocks on each day for the Small change condition.
**Table S3:** M200 (N2b): Paired samples *t*‐tests comparing the responses in the first and second stimulus blocks on each day for the Large and Small change conditions.
**Table S4:** M350 (P3b): Paired samples *t*‐tests comparing the responses in the first and second stimulus blocks on each day for the Large and Small change conditions.

## Data Availability

The raw data is not publicly available due to legal restrictions. The data that support the findings of this study are available upon reasonable request from Piia Astikainen (piia.astikainen@jyu.fi).
