# Supplementary material for: Rapid Mandarin Tone Learning in Passive and Active Listening: A Magnetoencephalography Study
Source: Eur J Neurosci. 2026 Mar 27;63(7):e70487. doi: 10.1111/ejn.70487 (PMC13031080; doi:10.1111/ejn.70487)
Supplement: Supplementary file 1 — Table S1: Behavioral results: Paired‐samples t‐tests comparing responses in the first and second stimulus blocks on each day for the Large change condition. Table S2: Behavioral results: Paired‐samples t‐tests comparing responses in the first and second stimulus blocks on each day for the Small change condition. Table S3: M200 (N2b): Paired samples t‐tests comparing the responses in the first and second stimulus blocks on each day for the Large and Small change conditions. Table S4: M350 (P3b): Paired samples t‐tests comparing the responses in the first and second stimulus blocks on each day for the Large and Small change conditions. [file EJN-63-0-s001.pdf]

Supplementary material

Rapid Mandarin Tone Learning in Passive and Active Listening: A Magnetoencephalography Study

Kaijun Jiang, Qin Li, Jari L.O. Kurkela, Simo Monto, Jarmo A. Hämäläinen, Xueqiao Li, Piia Astikainen*

Analysis of within-day changes in behavior and brain activity

Within-day changes in behavioral performance (accuracy and response time) and brain responses were examined using two-tailed paired-samples *t*-tests comparing responses between the two stimulus blocks on each day during active listening. Analyses were conducted separately for the Small and Large change conditions. Bonferroni corrections were applied to control for multiple comparisons. Within-day effects were not assessed for the passive listening condition because it contained only one stimulus block for each deviant type.

Results for the paired samples *t*-tests examining accuracy and response time differences between the two stimulus blocks within each day are presented in Table S1 (Large change condition) and in Table S2 (Small change condition). No significant differences were found.

Table S1. Behavioral results: Paired-samples *t*-tests comparing responses in the first and second stimulus blocks on each day for the Large change condition.

**Variable**	**Day**	***df***	***t***	***p***	**Cohen´s *d***	BF_{10}
Accuracy	Day1	8	1.72	0.492	0.57	0.94
	Day2	8	0.27	1.000	0.09	0.33
	Day3	8	0.99	1.000	0.33	0.48
	Day4	8	1.91	0.372	0.64	1.16
Response time	Day1	8	0.07	1.000	0.02	0.32
	Day2	8	0.75	1.000	0.25	0.41
	Day3	8	0.83	1.000	0.28	0.43
	Day4	8	2.10	0.276	0.70	1.43

Note. p-values are Bonferroni-corrected.

Table S2. Behavioral results: Paired-samples *t*-tests comparing responses in the first and second stimulus blocks on each day for the Small change condition.

**Variable**	**Day**	***df***	***t***	***p***	**Cohen´s *d***	BF_{10}
Accuracy	Day1	8	1.76	0.464	0.59	0.98
	Day2	8	0.07	1.000	0.02	0.32
	Day3	8	1.27	0.956	0.43	0.61
	Day4	8	0.80	1.000	0.27	0.42
Response time	Day1	8	1.70	0.508	0.57	0.92
	Day2	8	1.85	0.408	0.62	1.08
	Day3	8	0.18	1.000	0.06	0.33
	Day4	8	1.60	0.588	0.54	0.83

Note. p-values are Bonferroni-corrected.

Results of the paired-samples *t*-tests comparing M200 amplitudes between the two stimulus blocks within each day during active listening are presented in Table S3. No significant differences were found.

Table S3. M200 (N2b): Paired samples *t*-tests comparing the responses in the first and second stimulus blocks on each day for the Large and Small change conditions.

**Variable**	**Day**	***df***	***t***	***p***	**Cohen´s *d***	BF_{10}
Large change	Day1	8	0.90	1.000	0.30	0.45
	Day2	8	2.37	0.180	0.79	1.97
	Day3	8	0.05	1.000	0.02	0.32
	Day4	8	0.41	1.000	0.14	0.35
Small change	Day1	8	0.05	1.000	0.02	0.32
	Day2	8	1.36	0.844	0.45	0.66
	Day3	8	1.74	0.480	0.58	0.96
	Day4	8	0.86	1.000	0.29	0.44

Note. p-values are Bonferroni-corrected.

Results for the paired samples *t*-tests comparing in M350 amplitudes between the two stimulus blocks within each day during active listening are presented in Table S4. No significant differences were found.

Table S4. M350 (P3b): Paired samples *t*-tests comparing the responses in the first and second stimulus blocks on each day for the Large and Small change conditions.

**Condition**	**Day**	***df***	***t***	***p***	**Cohen´s *d***	BF_{10}
Large change	Day1	8	0.26	1.000	0.09	0.33
	Day2	8	0.01	1.000	0.01	0.32
	Day3	8	0.49	1.000	0.17	0.36
	Day4	8	0.76	1.000	0.25	0.41
Small change	Day1	8	0.50	1.000	0.17	0.36
	Day2	8	1.00	1.000	0.33	0.48
	Day3	8	0.17	1.000	0.06	0.33
	Day4	8	1.86	0.404	0.62	1.09

Note. p-values are Bonferroni-corrected.
